# Human Milk and Allergic Diseases: An Unsolved Puzzle

**DOI:** 10.3390/nu9080894

**Published:** 2017-08-17

**Authors:** Daniel Munblit, Diego G. Peroni, Alba Boix-Amorós, Peter S. Hsu, Belinda Van’t Land, Melvin C. L. Gay, Anastasia Kolotilina, Chrysanthi Skevaki, Robert J. Boyle, Maria Carmen Collado, Johan Garssen, Donna T. Geddes, Ralph Nanan, Carolyn Slupsky, Ganesa Wegienka, Anita L. Kozyrskyj, John O. Warner

**Affiliations:** 1 Department of Paediatrics, Imperial College London, London W2 1NY, UK; r.boyle@nhs.net (R.J.B.); j.o.warner@imperial.ac.uk (J.O.W.); 2 Faculty of Pediatrics, I.M. Sechenov First Moscow State Medical University, 119991 Moscow, Russia; aikolotilina@yandex.ru; 3 The In-FLAME Global Network, an Affiliate of the World Universities Network (WUN), West New York, NJ 07093, USA; diego.peroni@unipi.it (D.G.P.); albaboix90@gmail.com (A.B.-A.); peter.hsu@health.nsw.gov.au (P.S.H.); melvin.gay@uwa.edu.au (M.C.L.G.); Chrysanthi.Skevaki@uk-gm.de (C.S.); mcolam@iata.csic.es (M.C.C.); Donna.Geddes@uwa.edu.au (D.T.G.); gwegien1@hfhs.org (G.W.); 4 Department of Clinical and Experimental Medicine, Section of Paediatrics, University of Pisa, 56126 Pisa, Italy; 5 Institute of Agrochemistry and Food Technology, National Research Council (IATA-CSIC), 46980 Valencia, Spain; 6 Allergy and Immunology, The Kids Research Institute, The Children’s Hospital at Westmead, Sydney, NSW 2145, Australia; 7 Nutricia Research, 3584 CT Utrecht, The Netherlands; Belinda.vantland@danone.com (B.V.L.); johan.garssen@danone.com (J.G.); 8 Department of Paediatric Immunology, Wilhelmina Children’s Hospital, University Medical Centre Utrecht, 3584 EA Utrecht, The Netherlands; 9 School of Molecular Sciences, The University of Western Australia, Perth, WA 6009, Australia; 10 Institute of Laboratory Medicine and Pathobiochemistry, Molecular Diagnostics, Philipps University Marburg, University Hospital Giessen and Marburg GmbH Baldingerstr, 35043 Marburg, Germany; 11 Utrecht Institute for Pharmaceutical Sciences, Faculty of Science, Utrecht University, 3584 CG Utrecht, The Netherlands; 12 Charles Perkins Centre Nepean, University of Sydney, Sydney, NSW 2747, Australia; ralph.nanan@sydney.edu.au; 13 Department of Nutrition, University of California, Davis, CA 95616-5270, USA; cslupsky@ucdavis.edu; 14 Department of Public Health Sciences, Henry Ford Health System, Detroit, MI 48202, USA; 15 Center for Urban Responses to Environmental Stressors, Detroit, MI 48202, USA; 16 Department of Pediatrics, University of Alberta, Edmonton, AB T6G 1C9, Canada; kozyrsky@ualberta.ca; 17 National Institute for Health Research, Collaboration for Leadership in Applied Health Research and Care for NW London, London SW10 9NH, UK

**Keywords:** breastfeeding, human milk, allergy, allergic diseases, oligosaccharides, microbiome, cytokines, thymus

## Abstract

There is conflicting evidence on the protective role of breastfeeding in relation to the development of allergic sensitisation and allergic disease. Studies vary in methodology and definition of outcomes, which lead to considerable heterogeneity. Human milk composition varies both within and between individuals, which may partially explain conflicting data. It is known that human milk composition is very complex and contains variable levels of immune active molecules, oligosaccharides, metabolites, vitamins and other nutrients and microbial content. Existing evidence suggests that modulation of human breast milk composition has potential for preventing allergic diseases in early life. In this review, we discuss associations between breastfeeding/human milk composition and allergy development.

## 1. Introduction

Over the last few decades there has been a worldwide steady increase in the prevalence of allergic diseases [1]. Commensurate with a decrease in infectious diseases, allergy has become a considerable health/economic burden, most notably in relatively more affluent countries [2]. However, similar trends are starting to be seen in the developing world [3]. Explanations for this virtual allergy pandemic are not entirely clear but the “hygiene hypothesis” [4] remains the most widely quoted theory, explaining allergic disease rise as a mutually counter-regulatory interaction between the immune response to infection and that associated with allergy. Urban affluent lifestyles have been associated with significantly reduced infant exposure to bacterial infection and an altered commensal microbiome leading to a default allergic pattern of immune responses to common environmental ostensibly harmless antigens/allergens. Earlier birth order and/or fewer number of siblings, late or no attendance in day care facilities, and reduced exposure to pets [5] are among factors most commonly associated with allergic disease development. The apparent importance of rural environment exposure has been demonstrated in the study by Sozanska and co-authors [6], showing dramatic changes in community lifestyle leads to increased risk of allergy development. The accession of Poland to the European Union and changes in agricultural policies have resulted in an increase in prevalence of allergic diseases over an eight-year period. Within this timeframe, population contact with domestic animals and unpasteurised milk consumption has significantly declined while allergy rates have risen.

Although the “hygiene hypothesis” provides a mechanistically credible explanation for the rise in allergy prevalence, other societal factors have been brought forward, such as dramatic changes in dietary preferences over the past few decades. The less frequent consumption of fresh fruit, vegetables and fish has lowered fibre intake, and altered omega-3 and omega-6 polyunsaturated fatty acid (PUFA) ratios. Other environmental factors include lack of ultraviolet exposure leading to vitamin D insufficiency, greater exposure to air pollutants such as volatile organic compounds, diesel particulates and ozone, and even, increased exposure to chemical contaminants from packaged foods. Allergy is therefore, perceived as a “modern malady” prompting clinicians, researchers and policy makers around the globe to search for effective primary prevention [7]. Preventative strategies are particularly important for children at high risk of allergy development [8,9], with one or both parents being allergic [10]. It is suggested that the “window of opportunity” for allergy prevention is somewhere within the timeframe between conception and the first six months after birth [7,11,12]. As virtually all association studies do not discriminate between exposures of the mother during pregnancy, and/or lactation, or those directly affecting the infant, it is not possible to attribute a more exact timing of the “window”. It is perhaps more likely that a sequence of events during pregnancy and the early months of life combine to alter the risk of allergic sensitization and subsequent disease.

Human milk (HM) should be the main source of nutrition during a critical period of metabolic and immune programming, driven in part by its effects on intestinal function. Accumulated data suggests that a wide range of bioactive factors: such as proteins, polyunsaturated fatty acids, oligosaccharides, microbial content, metabolites, and micronutrients [13] present in HM can influence the infant’s gut immune maturation. Chronic allergic diseases are linked with the altered functioning of the innate and adaptive immune systems [14] and evidence suggests that it can be influenced using interventional strategies [15]. Recent research shows that various maternal exposures, such as immunisation, dietary patterns, vitamin D, ω-3 fatty acids and/or probiotics, may influence HM composition and thereby affect infant health. HM composition varies over time from delivery, within and between women, and even within the same feed, which may in part, explain some of the conflicting results of general observational studies regarding the provision of breastfeeding. Although HM constituents will be critical in influencing a range of other aspects of breastfeeding, such as its “exclusivity”, the close physical contact during nursing and time of weaning may also have important implications for health and development. However, results are inconsistent between studies, and there is no clear understanding of the pathways linking the intervention with effects on HM composition and health outcomes.

This review summarises existing evidence on breastfeeding and human milk composition in relation to allergic disease development.

## 2. Breastfeeding and Immunological Outcomes

Many aspects of breastfeeding can potentially influence its health effects [16]. These include duration of breastfeeding, maternal diet during lactation [17], and age at complementary food introduction [18,19,20], which can all differentially affect how breastfeeding may act on child health and immune development. Breastfeeding alters a child’s gut microbiome and subsequent immune development [21,22] and influences risk of respiratory infections through maternal antibody transfer [21]. It also impacts childhood nutrient intake such as vitamin D. The latter nutrient has been of particular interest because there are vitamin D receptors on many immune active cells and most notably on regulatory T-cells. Insufficiency is associated with reduced T-cell regulation of immune hyper-sensitive responses [23]. Data from some studies suggests that breastfeeding may impact immune organ functioning, with a difference in thymus involution seen between breastfed and formula fed children (discussed in more detail in Section 2.3).

It is well established that breastfeeding confers protection against both short-term adverse outcomes including reduced morbidity and mortality from neonatal infections) and long-term events including reduction in blood pressure, type 2 diabetes, increased IQ and better educational achievements in later life (even when adjusted for family socio-economic status [24]) [25]. A World Health Organisation (WHO) report suggests that there is a lower long term morbidity from gastrointestinal and allergic diseases in infants who were exclusively breastfed for 6 months in comparison to non-breastfed children [26]. Moreover, breastfeeding seems to play an important role at a time of complementary food introduction. Thus, during introduction of gluten into the infant diet it may reduce the risk of coeliac disease, suggesting important interactions between BM components, dietary antigens, and gut associated lymphoid tissue (GALT) [27]. However, this protective effect on coeliac disease remains uncertain, as studies have produced conflicting evidence [28]. Similar associations of reduced allergy in infants who have continued being breastfed during weaning have been reported [29]. Based on these data, current UNICEF and WHO recommendations are “every infant should be exclusively breastfed for the first six months of life, with continued breastfeeding for up to two years or longer” [30].

Despite some high-quality research, there is conflicting evidence on the protective role of breastfeeding in relation to many non-communicable diseases, including immunological (allergic and autoimmune) outcomes. It has been hypothesised that the mixed results may be in part due to variations in HM composition as it is known to contain a large variety of immune active components [13] which are present in differing concentrations [31]. Which factors are able to provide sufficient influence on short and long-term health outcomes in infants is still a matter of discussion, despite a number of studies attempting to address this question.

### 2.1. Importance of Breastfeeding Duration

When evaluating the relationship between breastfeeding duration and health outcomes it is important to have clear definitions for breastfeeding duration. It is usually defined as total breastfeeding duration, the time between birth and complete cessation of breastfeeding; while exclusive breastfeeding duration is the time between birth and first introduction of a non-breastmilk feed. Feeding expressed breastmilk, fresh or frozen, by bottle, and use of donor breastmilk, given directly or fresh or frozen by bottle, are variably included within these definitions of total or exclusive breastfeeding, depending on the focus of the research study. Use of the term exclusive breastfeeding is problematic in that it combines two separate interventions—timing of first solid (‘complementary’) food introduction, and use of a breastmilk substitute (formula milk). In general the evidence that early formula milk introduction is not optimal for development of infants health is stronger than the evidence that early complementary food introduction causes harm. It also depends on the type of allergy developed and exposure of allergens involved. This discrepancy has been highlighted by recent data showing that early complementary food introduction has defined health benefits—recent studies show that the phenomenon of oral tolerance induction, known for over 100 years to occur in animal experiments, also occurs in humans [7,29,32,33,34]. Oral tolerance occurs when early and sustained feeding of a food antigen reduces risk for developing food allergy to that antigen. This phenomenon has been shown to occur in humans for the two most common food allergies affecting young children: egg and peanut allergy [34]. However it is important to underline that tolerance development was only demonstrated in the per protocol and not ITT group in the latter study and further research is needed to make definitive conclusions.

This first sign that early introduction of complementary foods may be beneficial to infant health, suggests that future studies will need to more clearly distinguish timing of infant formula introduction and timing of complementary food introduction when evaluating relationships between exclusive breastfeeding duration and allergic disease risk.

### 2.2. Breastfeeding and Allergic Diseases

At the beginning of the last century, Grulee and Sanford suspected a link between HM substitute feeding and a higher incidence of eczema [35]. Since then many prospective and retrospective observational studies have tested breastfeeding associations with the onset of allergic disease, providing mixed results for eczema [19,20,21,36,37,38,39,40,41,42,43,44,45,46,47,48,49] sensitisation [21,37,42,46,47,48,49,50,51,52,53,54,55,56,57,58] and asthma [19,20,21,36,42,47,48,59,60,61,62,63,64,65,66]. Messages culminating from these studies range from a protective effect of breastfeeding [67], to a higher risk of atopy [68], or no significant effect [69]. Despite the conflicting evidence, several clinical societies have made recommendations regarding the duration and type of breastfeeding. As mentioned above, the WHO recommends exclusive breast feeding for at least 6 months in all infants with continued breastfeeding up to 2 years or longer if a mother wishes to do so [30].

The first efforts to systematically review existing evidence on breastfeeding associations with the selected eczema [70] and asthma [71], were made by Gdalevich and Mimouni two decades ago. Later, additional systematic reviews and meta-analyses were undertaken, assessing worldwide evidence [26,72,73], or focusing on data from developed countries [74]. The main challenge in the meta-analyses of these data was significant heterogeneity in the definitions of breastfeeding, which are not always consistent with WHO recommendations, and in phenotyping of for health outcomes. In the most recent systematic review which was published just two years ago [75], Lodge and colleagues reported on 4 different definitions of eczema, food allergy and asthma, and 3 definitions of allergic rhinitis used across studies [75], with differing breastfeeding exclusiveness and duration creating even more uncertainty.

Assessment of breastfeeding’s potential to prevent allergic disease in observational studies is not an easy task as several factors, such as socioeconomic status, positive allergy family history, early exposure to pets and timing of solid food introduction, alongside variations in HM composition, are all sources of bias. Prospective randomised studies are needed to provide solid evidence of causal relationships, however such studies would be unethical. The sole large randomized controlled trial used an innovative approach in a country with a very low breast feeding rate and investigators randomised mothers to a breast feeding promotion group or continued standard practice. The intervention significantly increased breast feeding rates and facilitated evaluation of the breastfeeding associations with health outcomes in Belarus [76]. There was a reduced risk of early eczema (Odds Ratio (OR) 0.54, 95% CI 0.31–0.95) with breastfeeding but no long-term protection against eczema, allergic rhinitis and asthma at 6.5 years of age, despite the long duration and exclusivity of breastfeeding observed in this trial [77]. However, the extent to which WHO recommendations delayed introduction of non-milk food sources at a critical period where tolerance induction may be important to prevent allergy is uncertain [78].

#### 2.2.1. Eczema

Most of the studies assessing breastfeeding impact on eczema development come from the cross-sectional studies or birth cohorts. The major weakness of collected data is related to a long retrospective recall period and lack of adjustment for potential confounding factors, such as allergy family history [75]. Authors of The International Study of Asthma and Allergies in Childhood (ISAAC), a large observational study assessing more than two hundred thousand children worldwide, failed to find evidence of a breastfeeding protective effect on eczema development at 6–7 years of age (OR 1.05, 95% CI 0.97–1.12), but reported some protection against severe eczema (OR 0.79, 95% CI 0.66–0.95) [79]. Outcomes of a systematic review and meta-analysis, covering literature up to 2014, suggested that children below 2 years of age who were exclusively breastfed for more than 3–4 months were are at lower risk (OR 0.74, 95% CI 0.57–0.97) of eczema development; however, this protective effect was no longer evident after the age of 2 (OR 1.07, 95% CI 0.98–1.16) [75]. The authors highlighted a potential high risk of bias from smaller studies showing more significant protective effects.

#### 2.2.2. Food Allergy

Studies assessing the association between breastfeeding and food allergy contribute conflicting results, with some cohort studies reporting a reduced risk of food allergy development in a general population [80,81] and in high risk children [82], with others suggesting a greater risk after breastfeeding [83,84]. The most recent meta-analysis showed no statistically significant association between breastfeeding and food allergy development (OR 1.02, 95% CI 0.88–1.18). Assessment of food allergy is not straightforward in the context of a clinical trial, as the gold standard for confirming the diagnosis is the double-blind food challenge, which is not always a viable option for study participants. In many studies, a combination of a clinical history and skin prick test (SPT) or serum IgE testing is used as surrogate markers of a diagnosis of food allergy with inevitable high heterogeneity. Hence, the primary goal for future research should be harmonization of the outcome definition [75]. Recent clinical trials showing benefits in early food introduction (from 3 to 4 months of age), in parallel with breastfeeding, may indicate a worthwhile strategy to decrease risks of food allergy development. This has been driven by recent studies, such as the Learning Early About Peanut Allergy (LEAP) and Enquiring About Tolerance (EAT) trials [29,33], suggesting that in some children, early introduction (before child age 6 months) of peanut and/or egg protein reduces the risk of allergy to these foods [7].

#### 2.2.3. Asthma

More than 15 years ago, Gdalevich and Mimouni reported a link between breastfeeding and lowered asthma prevalence in children (OR 0.70, 95% CI 0.60–0.81) [71]. This association has been further confirmed in two subsequent meta-analyses (OR 0.78, 95% CI 0.74–0.84] [73] and OR 0.88, 95% CI 0.82–0.95 [75]). Biological plausibility or coherence in published evidence for a role of breastfeeding in protecting against asthma development includes its demonstrated benefit in reducing the number of respiratory tract infections in early infancy, especially among infants in middle- and low-income countries [75]. In addition, exclusive breastfeeding reduces the duration of hospital admission, risk of respiratory failure and the requirement for supplemental oxygen in infants hospitalized with bronchiolitis [85,86]. Some of the described protective effects may be mediated through an antiviral mechanism or non-specific enhancement/maturation of the infant immune system.

However, there is significant heterogeneity (study design, outcome definition, country development) between studies reporting an inverse association between breastfeeding infants and asthma development. Some of the differences can be explained by variations in the definitions for breastfeeding exclusivity and duration, and methods to diagnose asthma in children [87]. It is known that many infants who wheeze in the first years of life do not develop asthma in later life [88], but wheeze is often used as the diagnostic marker of asthma. There are several notable large prospective birth cohorts, such as Avon Longitudinal Study of Parents and Children (ALSPAC) [50], Prevention and Incidence of Asthma and Mite Allergy (PIAMA) [89] and the cross-sectional International Study of Asthma and Allergies in Childhood (ISAAC) [90] study, with data from these studies considered of higher quality [75]. The protective effects of breastfeeding on asthma are more apparent in recent studies, perhaps due to improvements in methodology [73,75]. It is worth noting that subgroup analysis shows a greater protective effect of breastfeeding in middle to low income countries where allergy is less common [75]. It seems likely that the major effect is on respiratory infection induced wheeze rather than atopic asthma. Future studies will need to phenotype and endotype asthma more precisely.

### 2.3. Breastfeeding, Thymus and Immunity

The thymus is an essential organ for generation of T cell immunity and tolerance. Lymphoid progenitors from the bone marrow migrate to the thymus, where a series of stringent positive and negative selection processes take place [91]. These processes are important for the production of functional T cells, which are able to recognize and respond to foreign/microbial antigens presented by the MHC in the periphery, but also Foxp3+ regulatory T (Treg) cells, which mediate immune tolerance to self and a variety of self and foreign antigens [92]. Not surprisingly, thymic aplasia as seen in DiGeorge syndrome is associated with immune deficiency and immune dysregulation [93]. Furthermore, in all vertebrates the thymus naturally shrinks in size with age. This process of thymic involution is poorly understood to date [94] but impacts directly on thymic output [95].

Thymic size can also be influenced by a variety of factors. Prenatally, maternal factors such as preeclampsia has been associated with reduced thymic diameter [96], although the mechanism and the consequences of this need to be further investigated. Postnatally, various events such as acute stress are known to reduce the thymic size [97].

Breastfeeding on the other hand, has been associated with increased thymic size. At 4 months of age, the thymus size (as assessed by ultrasound) in exclusively breast-fed infants was more than double the size of formula fed infants, an effect that persisted at least until 10 months of age [98]. A further study revealed that persistent breast feeding between 8 and 10 months also correlated with increased thymus size in a “dose dependent” manner [99]. Although the immune implication of this remains unclear, a subsequent study showed a correlation between breast feeding and peripheral CD4 and CD8 T cell counts and proportion [100]. The importance of thymic tissue for T cell immunity is further supported by a study showing that partial or total thymectomy in infants undergoing cardiac surgery was associated with lower T cell numbers and immunoglobulin levels later in life [101]. The mechanism by which breastfeeding may influence thymus size is unclear. However, one study conducted in rural Gambia suggested that the reduced thymic size and output in exclusively breastfed infants born in the “hungry season” compared to “harvest season” was associated with reduced Interleukin 7 (IL7) levels in the breast milk [102]. As IL7 is critical for thymopoiesis [103], it seems plausible that this cytokine may influence thymic size. However, other breast milk cytokines and metabolic components need to be considered as well.

In addition, breast milk is known to shape the infant’s gut microbiome [104]. The gut microbiome is the main source of bacterial metabolites such as short chain fatty acids, which have been shown to play a central role in T cell development and differentiation [105]. Hence, a mechanistic explanation implicating a beneficial role of breastfeeding on the infant’s gut microbiome may be an alternative explanation for enhanced thymic size in breastfed infants.

Overall, evidence suggests that breastfeeding influences thymic size. However, evidence is lacking regarding the mechanism and the immune impact of this observation. Future longitudinal cohort studies are required to address this. These studies should include good measures of thymic output such as assays of T cell receptor excision circles (TREC) and thorough immune phenotyping of T cell subsets, gut microbiome profiling as well as good clinical data on immune outcomes.

## 3. Human Milk Composition and Allergy

Human milk is the earliest and should be the only source of nutrition during first few months of life, a crucial period for infant immune system development and metabolic programming for lifelong health and development. Many biologically active components are found in HM, and there is some evidence, arising from the studies in humans, suggesting maternal exposures can change both HM composition and subsequent infant health outcomes [78,106,107,108].

### 3.1. Human Milk Immunological Composition

Variations in breast milk immune composition (and the infant’s response to HM immune constituents) may also explain some of the conflicting results of studies evaluating whether prolonged exclusive breast-feeding can prevent allergic disease [109,110]. Human milk is a “soup” full of immune active factors, including leukocytes (polymorphonuclear neutrophils, monocytes/macrophages, lymphocytes), which potentially may influence immunological outcomes in infancy and early childhood. It contains over 250 potentially immunologically active proteins, including a wide variety of cytokines, inflammatory mediators, signalling molecules, and soluble receptors [13], as well as prebiotic oligosaccharides: polyunsaturated fatty acids (PUFAs) [111]: and a diverse microbiome [112], all of which are involved in complex interactions which could influence immune outcomes.

Colostrum (early human milk, produced during the first days of life) is very rich in immunologically active molecules that are present in much higher concentrations than mature HM [106,113,114,115,116]. The levels of growth factors in colostrum decline very rapidly, which may be partially explained by increasing dilution, as in the first days of life the infant’s volume requirements are low [116]. As HM matures, the relative concentrations of the immunologically active molecules decrease as the volume and nutritional requirements of the infant increase.

There is only limited literature on the relationship between maternal diet (including intervention trials), human milk immunological composition, and allergy development [117,118]. The main studies are summarised in Table 1 and Table 2.

#### 3.1.1. Immune Composition and Allergy

Among the immunological markers assessed in HM, TGF-β is probably the most studied to date. The systematic review by Oddy and Rosales assessed relationships between TGF-β in human milk and immunological outcomes in infants and children [140]. Two-thirds the studies selected for this review found an association between higher TGF-β1 or TGF-β2 levels in colostrum or mature milk and reduced risk of atopic outcomes in the infant. The authors suggested that TGF-β found in human milk may play a role in homeostasis maintenance in the intestine, regulating inflammation and subsequently promoting oral tolerance which may reduce the risk of allergy development [140].

A few studies focused on eczema, found increased TGF-β1 and/or TGF-β2 in HM associated with this skin disease onset in infants [122,126,130,139]. However, contrasting results of other studies do not allow final conclusions on the influence of TGF-β on eczema development [132,135,137,138]. Oddy and co-authors reported increased TGF-β1 levels in breast milk to have some protective effect against wheeze development in infancy [133] but this conflicts with two other large cohort studies [135,138]. As it is assumed that TGF-β has biological relevance and is active in the infant gut [141], these results suggest that TGF-β plays an important role and may be a missing component of progression from allergic sensitisation to allergy disease in early life, but inconsistency in results prevents us from making any definitive statements. Differences in the outcomes can be affected by the stage of lactation when samples were collected.

Another immune active molecule that is of interest is soluble CD14, a bacterial pattern recognition receptor for cell wall components such as lipopolysaccharide. It is primarily expressed on the surface of monocytes, macrophages and neutrophils as membrane CD14 [142,143] but is also found in HM in its soluble form—sCD14. In all the studies levels of sCD14 in HM were very high as this immune active molecule is amongst those immune factors actively excreted into HM. CD14 may play an important role, providing protection against subsequent allergy manifestation [144,145,146]. More than a decade ago, Jones and co-authors showed that low sCD14 levels in mature milk were associated with eczema development [131] and then Savilahti reported similar trends for colostrum [134]. Later studies, however, failed to reproduce these results and did not report any protective effect of this soluble receptor on eczema [135,137]. The conflict between the outcomes of the studies may be a consequence of a difference in CD14 genotype with breastfeeding being associated with a decreased risk of atopic sensitisation in children with a CT/CC genotype [52]. We now recognise eczema as a consequence of genetically determined skin barrier defects with allergy being a likely secondary outcome. Phenotyping and genotyping eczema in relation to breast feeding therefore becomes a priority.

There is a general agreement between studies suggesting that levels of other human milk immune active molecules, cytokines in particular, are not associated with atopy and/or allergy development in early life [126,132,135,137]. The only outlying results come from the recent paper by Jepsen and co-authors, suggesting that high levels of IL1β in mature milk are associated with lower risk of eczema by the age of 3 [58] and Jarvinen et al. showing that networks of pro-inflammatory and regulatory cytokines in HM are associated with tolerance to cow’s milk [147]. As many cytokines exist in very low concentrations in HM, the sensitivity of the assays is critical and many studies report a high proportion of undetectable levels in their samples [58,148,149]. This may explain lack of conclusive data on HM cytokines association with immunological outcomes. Furthermore, if there are only trace levels of these mediators they are unlikely to have significant biological activity. Future studies will need to assess biological activity alongside assays of concentrations.

Most of the studies were aimed at allergic sensitisation, eczema, early wheezing and/or asthma and allergic rhinitis development as the main phenotypic outcomes which allow for some comparison. However, significant methodological heterogeneity between the studies, especially with regards to the stage of HM collection and outcome definitions, are the main obstacles on the way to any meta-analysis of up to date data in this field. Despite these difficulties it is apparent that certain factors of interest in HM may play a role in allergic sensitisation and/or allergy prevention. The most promising HM components are TGF-β, sCD14, and particularly their relationship with HM oligosaccharides (HMOs) and microbiome, interactions which have not been extensively studied and may represent a prime area for future research. In view of the large number of potentially immune-active constituents in breast milk, investigation of only a limited range of constituents may well produce conflicting results. There is a lack of studies, attempting to assess HM as a whole, rather than focusing on single components. In other words, the “soup” is likely more important than individual ingredients. 

#### 3.1.2. Potential for Immunological Composition Alteration via Dietary Interventions

Given the observations discussed above there is the intriguing possibility for interventions which modify maternal immunity to impact infant immune responses and allergic disease in offspring [131,134]. With the development of the ‘hygiene hypothesis’ many focused their research on the protective effects of environmental exposures during pregnancy and early life, during a period of time when infant gut colonization and maturation of the immune system takes place. Despite a number of birth-cohort studies, the ability to change human milk composition remains a “grey area” in existing knowledge and more hypothesis driven research is required before large population intervention trials can begin.

Existing data provides evidence that HM composition is highly variable within the same individual and between women. It has been shown that maternal lifestyle (dietary habits, physical activity, place of residence) can have a significant influence on HM biologically active components [106,107,108,116]. These findings have motivated a number of intervention trials aiming to prevent allergy development in early infancy.

There are many trials of probiotic administration, as single-entity products of a specific strain or mixtures, in the prevention of allergy development, with cumulative meta-analytic evidence suggesting some protection against eczema [150]. Prescott et al. observed higher levels of TGF-β1 and IgA in human milk of mothers receiving B. lactis HN019 probiotics, and higher IgA levels alone in those receiving *L. Rhamnosus* HN001. In contrast probiotic supplementation did not seem to have an effect on the rest of BM immunological profile (IL-13, IFN-γ, IL-6, TNF-a, IL-10 and sCD14) [123]. Two other studies of probiotic use during pregnancy reported no effect on TGF-β levels in HM [124,151] and they were in opposition to findings by Rautava and co-authors [152]. Heterogeneity of methods again confounds attempts at meta-analysis.

Another potential intervention approach is the use of prebiotics. Prebiotics are non-digestible food components that may confer benefit by providing the substrates for normal bacterial growth the gut. It is more common now to see prebiotics added to formula milk. It is unclear whether prebiotics are capable of modifying HM composition or influencing subsequent allergy development in both high risk and general populations [153].

PUFAs (e.g., ω-3 and ω-6 fatty acids) are an essential part of HM composition and, as a logical investigation, researchers have attempted to influence PUFA levels in HM by means of intervention, selecting fish oil or whole fish as a main source of PUFA. Some of these studies also evaluated HM immunological composition. Data from several intervention trials showed no apparent evidence for the impact of fish consumption on immune active molecules in HM [119,120,121]. Another source rich in ω-3 and ω-6 fatty acids is blackcurrant seed oil. A Finnish study reported lower levels of IL-4 and increased IFN-γ in HM following black currant seed oil consumption, with no differences in IL-5, IL-10, IL-12 and TNF levels, in comparison to an olive oil fed group [128].

Overall, there is some evidence that probiotic [123,124,152] administration to pregnant and lactating women, or a diet with a high fish intake [121] alters breast milk immune composition. Although the specific changes identified are not always correlated with clinical outcomes, maternal supplementation during pregnancy and lactation to enhance human milk “quality” may have a beneficial influence on health outcomes, and modulation of breast milk composition is one possible mechanism [154] (see Table 2).

### 3.2. Human Milk Oligosaccharides 

#### 3.2.1. The Fascinating Complexity of Human Milk Oligosaccharides

Unique to HM is the complexity and abundance of HMOs consisting of both short-chain as well as long-chain oligosaccharide structures in a unique ratio based on molecular size (roughly 9:1 respectively). Together with specific metabolites derived from bacterial fermentation, the HMOs play a key role in microbiome development and building a healthy immune system, creating a fit and resilient immune system in early and later life [155]. It is important to realize that the complex HMO composition is determined by genetic polymorphisms and activity of the secretor fucosyltransferase2 gene (FUT2), the *Lewis* gene (FUT3), and is regulated by glycosyl-transferases within the mammary gland. Differences in genetically determined glycosyl-transferase patterns affect HMO amount and composition between mothers and during lactation [156]. The presence or absence of α1,2-linked fucosylated epitopes in secretions, including saliva and milk, defines secretor and non-secretors respectively. Consequently, the secretor-phenotype distribution differs among populations [157,158]. The provision of secretor type related complex mixtures of HMOs, have been associated with a direct protection against infections [159] and may be linked to a reduction in allergic disease incidence in breast-fed infants later in life [46].

The basic HMO structure is fucosylated and/or sialylated, resulting in respectively neutral and acidic oligosaccharide structures within short- as well as long-chain structures. In addition to the inter-individual genetic variation, the total HMO concentration varies during lactation which normally provides the optimal needs over time. Colostrum contains approximately 20–25 g/L HMOs, whereas mature HM has declining HMO concentrations to 5–15 g/L [160,161]. 2′-Fucosyllactose (2′-FL) is a disaccharide which is thought to be the most abundant oligosaccharide with a concentration ranging from 0.06 to 4.65 g/L [157,158]. Each HMO is structurally unique and effects of individual structures may not be universal to all HMOs, therefore understanding the balanced complex mixture is of considerable importance.

Although the protective capacity of HM against infections within infants is clearly observed, the possible benefit for the prevention of immune related disorders such as allergy remains controversial [135,162,163]. Any discordance between the early developmental requirements for an infant’s immune development and the dynamic nature of HM constituents may possibly contribute to the development of allergic diseases [162]. Whether observed effects are derived from direct interaction with immune cells or indirectly through the alterations in microbiome composition and change in derivatives thereof remains unknown. It is clear however, that the microbiota composition and activity can have an influence on the development of allergy, more specifically regulatory T cell development is strongly influenced by the microbial composition, and therefore subject to modulation by dietary intervention and specific oligosaccharides [164,165,166]. Several studies have shown that the composition of the gut microbiome differs significantly between those with allergy and/or allergic disease and those without [167,168,169,170,171]. How a microbiome composition becomes dysbiotic and thereby leads to the development of immune related disorders such as allergy is hitherto not fully understood; however, it is thought that early-life ecological succession of mucosal colonization occurs concomitantly with development, expansion, and education of the mucosal immune system [172]. Indeed, gnotobiotic mouse studies have demonstrated that there is a critical window of time for immune development, after which intestinal immune development cannot be fully achieved [173,174,175].

#### 3.2.2. Shaping the Microbial Balance in Early Life

The question of how optimal early-life microbial ecological succession occurs is a topic of intense interest. Once HMOs are formed, only those bacteria that possess the necessary enzymes (incl. glycosyl hydrolases) can cleave and utilize these oligosaccharides [176]. Members of the *Bacteroidaceae* and *Bifidobacteriaceae* families have been shown to consume HMOs, including several *Bifidobacteria* which have the sialidases and glycosidases necessary to internalize and catabolize HMOs [177,178,179,180,181]. What this means is that for breast-fed infants, *bifidobacteria* have the capability to preferentially colonize the infant GI tract by the third month of life [177]. In addition, it was recently shown in a mouse study that the combination of *B. infantis* with HMO decreased GI inflammation and permeability [182]. Other mouse studies revealed that oral administration of *Bifidobacteria* is able to modulate inflammation associated with allergy [171,183,184]. However, the total HM oligosaccharide composition is likely to be very important and it should be realized that individual oligosaccharides in HM might have their own unique function on microbes, immune cells and epithelial cells.

Given that maternal secretor status impacts the bifidobacterial community structure of the infant gut [185], it can be hypothesized that a combination of HMOs with specific bacteria are able to modulate gut immunity and gut integrity. Additional roles of fucosylated and sialyated HMOs are related to the common structural motifs they share with glycans on the gut epithelia that are known receptors for pathogens. It is thought that HMOs competitively interact with pathogens, preventing adhesion and biofilm formation on the gut epithelium [186,187,188]. Together with their ability to only be fermented by specific bacteria, HMOs therefore play an important role in shaping early gut microbial succession. The nature of this succession, and exactly how different oligosaccharides function in this context, are questions that remain to be elucidated.

As previously discussed, there have been conflicting reports regarding the relationship between breastfeeding and development of allergy [19,117,189,190], and it may be that it is the combination of oligosaccharides and bacteria that shape immunity. Indeed, it was recently reported that infants born by caesarean section with a high risk of allergies had a lower risk of IgE-associated eczema at 2 years, but this association was not observed at 5 years [46]. In addition, prebiotic oligosaccharides together with *Bifidobacteria* have recently shown in caesarean-delivered infants to be able to modulate the microbial composition which was associated with the emulation of the gut physiological environment observed in vaginally delivered infants [191,192]. Moreover, epidemiological studies have frequently shown that there is a clear associational link between perinatal factors, such as breastfeeding, caesarean delivery, and antibiotic use, and the programming of intestinal inflammatory disorders. However, more work needs to be done to fully understand how HMOs and allergy development are related.

#### 3.2.3. HMOs Are Directly Involved in Early Life Immune Development

How the complete mixture of and/or specific HM oligosaccharides are able to beneficially regulate gut microbiota composition, maintain gut integrity, and most importantly, enhance mucosal immunity to establish a balanced immune development is not completely understood. Because of the multiple different structures within authentic HMOs, several distinct receptors and pathways are thought to play an important role within the direct immune modulating role of HMOs. Direct interaction has recently been shown (by using glycan microarray technology) between glycan-binding proteins expressed on the epithelial cells and cells of the innate immune system to specific HMOs. For instance, 2′-Fucosyllactose and 3-fucosyllactose were shown to bind human DC-SIGN (Dendritic cell-specific intercellular adhesion molecule-3-grabbing non-integrin), a C-type lectin receptor present on the surface of both macrophages and dendritic cells. Moreover, the involvement of a set of glycan binding receptors including the C-type lectin receptors and Toll-like receptors (TLR) has been identified [193,194,195]. The direct binding of 2′FL to human DC-SIGN has been shown to be fucose-specific and DC-SIGN signaling seems to be influenced, leading to alteration in pro-inflammatory cytokine response in a TLR specific fashion [196,197]. In addition, it has long been known that human galectins expressed by intestinal epithelial cells also interact with oligosaccharides [198]. However, the exact mechanisms of how HMOs are able to alter the biological function of these human cells are still unknown.

Only a few limited studies have focused on the immune-modulatory effects of individual HMOs within infants and in animal studies, and have suggested anti-inflammatory and immune regulatory potential, but the mechanism by which specific HMOs may influence the risk for allergy development is currently not known [199]. Supplementation of the diet with 2′FL or 6′SL did not show any effect on the levels of allergen specific Immunoglobulin (Ig)E or IgG1 in sensitized or challenged mice. Dietary supplementation with specific oligosaccharides providing some of the functional benefits of HMOs, have been shown to reduce the risk of developing allergies in infants [200,201]. Recent data suggest that the onset of IgE-associated allergic manifestations, (but only in infants with a high hereditary risk for allergies and born by C-section) might be associated with FUT2-dependent oligosaccharide composition in breast milk consumed by these infants [46]. These mechanisms collectively include but may not be limited to the pathogen decoy capacity of specific HMOs, the prebiotic effect on the microbiome composition, the modulation of the SCFA production which in turn supports the barrier integrity and/or through direct immune modulatory functions [202]. However, further clinical studies are needed to support either one of these mechanisms to identify the full potential of HMOs within the early life immune development.

Within the last few years an interesting increase in understanding and knowledge regarding the presence and effects of HMOs and composition has been achieved. Consequently, with expansion of these studies and progress in biotechnology, the potential of adding HMOs to the complex mixture of prebiotic oligosaccharides in infant formulas are increasing. However, in order to decide which to add, in which concentration, composition and combination to prevent and treat allergy development in early life, as well as later in life, clearly needs additional study.

### 3.3. Human Milk Microbiota

Early microbial colonization is essential for infant’s metabolic and immunological development [203]. Cumulative evidence suggests a direct link between microbial colonization and the risk of non-communicable diseases in later life, including allergies [204,205]. After birth, the transfer of microbiota continues during lactation, and is considered to be the cause of differences in gut microbiota between exclusively breastfed and formula fed infants during the first months of life [206]. In the recent years the presence of a HM microbiome has been confirmed, with a variety of microbes and their associated genes and antigens transmitted to the infant during breastfeeding [112]. Available data show that HM contains approximately 10^3^–10^5^ viable bacteria per mL [207,208].

Initially, the presence of microbes in human milk was evaluated by use of culture-dependent techniques and isolates belonged to *Staphylococcus*, *Streptococcus*, and *Lactobacillus* and *Bifidobacterium* species, which have been used as probiotics in intervention trials [209]. With the development and application of culture-independent techniques including next-generation sequencing, it has become clear that HM contains a much more diverse variety of bacteria, including other lactic acid bacteria, such as *Enterococcus, Lactococcus* and *Weissella*; typical inhabitants of the oral cavity, such as *Veillonella* and *Prevotella;* bacteria usually found in the skin, like *Propionibacterium,* and other Gram negatives, e.g., *Pseudomonas*, etc., [112,210,211,212]. In a recent systematic review, a core of predominant organisms was described, which includes *Staphylococcus, Streptococcus* and *Propionibacterium* [213]. These genera are universally predominant in human milk, regardless of different potential confounding factors, such as sampling, geographic location or analytical methods [213].

There is still scarce information about the influence of environmental and perinatal factors on HM microbiota composition [214]. Some studies reported that geographical location [112,204,206,215], delivery mode [207,208], maternal body mass index (BMI) [211,216] or antibiotic intake [217] would have an impact on HM microbiota. However, others did not find similar effect with regards to other perinatal factors [218]. Furthermore, an imbalance in the normal bacterial composition of HM can lead to the overgrowth of specific opportunistic pathogens and lead to mammary infection, such as lactational mastitis [219,220].

The origin of HM bacteria is currently unknown. A number of hypotheses have been proposed: (1) human milk microbiota could derive from the mother’s skin, and the infant’s oral cavity during suckling; (2) an internal route, the “entero-mammary pathway” has been proposed and suggests that bacteria from maternal gut could be taken up by immune cells and transported via blood stream or lymphatic system to the mammary gland [221]; (3) specific microbes were detected in the human breast tissue, which may also supply microorganisms to the milk [222,223].

Human milk microbes hypothesised to play a key role as early gut colonizers, likely contribute to the immune system development and maturation [224,225]. Alterations or divergent antibodies/microbiota transferred via HM may affect an infant’s immune development. Lower proportions in the *Bifidobacterium* genus have been observed in HM from allergic mothers [226]. The gut microbiome from allergic children also differs from non-allergic in composition and diversity [226]. Recently, altered immune responses towards gut microbiota were observed as early as 1 month postpartum, in exclusively breastfed children who subsequently developed allergies [227]. Relationships between HM components (HMOs, fatty acids, immunological constituents, etc.) and allergy development in infants have been recently receiving increased attention [46,60,139,228,229,230]. Several studies reported that allergic disease and asthma are less common in children exposed to unpasteurized cow’s milk (CM), which is a source of viable microorganisms [231]. Therefore, bacterial communities of HM could also be taking part in the protection of infants against allergic diseases, acting as a natural probiotic, and this requires further elucidation. However, unpasteurized CM also contains many immune active constituents with close sequence homology to those found in HM. These could also potentially explain the benefits of raw CM. Existing data suggests that some *Lactobacillus* and *Bifidobacterium* strains have been linked to allergy protection, in particular against eczema [150]. It is worth noting, however, that eczema is not synonymous to allergy, as discussed in other sections of the manuscript. This highlights a need in precision of outcome definitions alongside pheno- and endo-typing infant outcomes. As these genera can be found in HM, it is, therefore, plausible that their transfer to the infant during breastfeeding could provide immunological protection, although more work is needed to confirm this link.

The potential protective effect of HM bacteria against allergic diseases development has not been properly studied and future research should also investigate HM bacterial recognition by the immune system. Better knowledge would help to understand the importance of maternal transference of altered immune responses towards microbiota during breastfeeding, and their potential influence on allergy development during infancy. However, it is rather difficult to establish causal relationships between HM microbiome and its role in protection against allergic diseases. It is impossible to rule out the probability of an epi-phenomenon, and future research should tackle cause-effect relationships. Further analysis based on state-of-the-art, next-generation sequencing methods will be crucial in understanding the association between bacterial diversity inherited through breastfeeding and an infant’s potential allergy development.

In clinical trials, oral administration of bacterial strains to lactating mothers showed modulatory effects both on human milk composition and on the infant’s gut. It was shown that *Lactobacillus reuteri* intake led to its detection in the mother’s milk and infant faeces [210]. Similarly, another study studied the effect of supplementation with *L. rhamnosus* to reduce the risk of allergy development when given to women during pregnancy and lactation [232]. Probiotic intake during pregnancy and lactation also induced specific changes in the infant *Bifidobacterium* colonization and influenced HM microbiota composition compared with those receiving placebo [233]. Recently, the effects of perinatal probiotic supplementation on the HM composition have been reinforced, leading to changes in its microbiota, including *Bifidobacterium* and *Lactobacillus* sp., and also functional components of HM, such as oligosaccharides (HMO) and lactoferrin [234].

Protective effects of certain *Lactobacillus* and *Bifidobacterium* strains on eczema development have been previously reported [235,236]. Their ability to provide protection against other allergic diseases has also been described, although results are conflicting and existing evidence does not support their use for allergy prevention. The beneficial effect on eczema has been proved [150], but the causality is still unclear. As eczema is a consequence of a skin barrier defect, the possibility of protection due to direct effects of short-chain fatty acids on skin rather than immune modulation cannot be excluded. If strong relationships between specific HM microorganisms and allergic diseases are further confirmed, prebiotics and probiotics could be used to improve HM composition and infant microbiota modulation.

### 3.4. Human Milk Micronutrients

While breastfeeding is recommended as the sole source of infant nutrition up to 6 months of age by WHO [237], there are caveats that an adequate maternal diet is required in conjunction with sufficient volumes of milk that can be transferred to the infant [237]. Lactating women and infants have a greater physiological demand for micronutrients and are therefore at higher risk of adverse consequences with insufficiency. Despite HM containing a multitude of micronutrients that are the infant’s sole source in early life, comprehensive methodical research has not been carried out in this area [238]. Further, many HM micronutrients differ between women, such as Vitamin A and group B vitamins, which are influenced by maternal dietary intake (Table 3). Owing to this variation and the limited number of studies that often use small participant numbers, frequently suffer from lack of control for stage of lactation, fail to record maternal supplementation, and have inconsistent sampling, robust reference ranges for HM micronutrients do not exist. To add fuel to the fire, various methods have been employed such as microbiology and radioisotope dilution with the recent addition of chromatography, coupled with UV, fluorometric and mass spectrometry detection making comparisons even more challenging. Only recently has there been a concerted effort to shed light on questions such as variation within feeding, circadian rhythms and the impact of maternal supplementation. This lack of research likely explains conflicting results and has subsequently hampered the determination of recommended daily intakes for infants [239]. The other potential explanation is failure to consider the timing of deficiencies. Transfer of nutrients to the foetus during pregnancy is likely equally, if not more important, than HM composition. During the first trimester of pregnancy, programming of growth trajectories will have a profound effect on foetal and infant requirements for micronutrients. Keeping in mind the “Developmental Origins of Health and Disease” (DOHaD) hypothesis, which suggests fetal developmental ‘plasticity’ and discordance between intra- and extra-uterine exposures produces the greatest adverse effects [240].

#### 3.4.1. Vitamin A

A number of HM vitamins are influenced by maternal diet including vitamin A, which plays a major role in both growth and immune function. In a small study of lactating Bangladeshi women (*n* = 18) intensive sampling showed that the most appropriate sample should be taken from a pumped volume from a full breast and that there was a small but significant circadian variation that disappeared when milk fat was accounted for. Further, vitamin A content increased significantly with acute supplementation [254]. Vitamin deficiencies in the infant included adverse outcomes such as severe respiratory and gastrointestinal infections, as well as increased morbidity and mortality [267]. In a mouse model, maternal supplementation during lactation prevented allergic airway inflammation and had a protective effect on oral tolerance induction [268]. This finding is consistent with a meta-analysis of human studies that shows dietary intake of vitamin A to have either a beneficial association in asthma prevention or no association [269]. In contrast, direct neonatal supplementation in human neonates appears to increase the risk of atopy and wheezing, particularly in females [270]. It is speculated that HM borne vitamin A reduces allergy via promotion of intestinal crypt development and a reduction of gut permeability without impacting the digestion of milk [16,268]. Future studies will serve to shed light on the protective mechanisms of HMvitamin A.

#### 3.4.2. B Vitamins

In general, group B vitamins concentration of HM is also strongly related to maternal intake and levels respond to dietary supplementation [271,272]. Levels of HM B vitamins are based on samples from women in established lactation, as thiamin, vitamin B-6, and folate are lower, and vitamin B-12 higher in the first few weeks of lactation (transitional milk) [272], whereas, in established lactation the levels of all B vitamins remain relatively stable [246]. Importantly, maternal depletion impacts infant status to varying degrees depending on the vitamin and the levels of the vitamin. Further, complicating the picture is the lack of global documentation on the prevalence of HM vitamin B deficiency.

Studies investigating relationships between vitamin B and infant allergy are also scant with one study showing no relationship between wheeze or eczema in infants (16–24 months) and maternal intake of folate, vitamin B12, vitamin B6, and vitamin B2 during pregnancy [273].

#### 3.4.3. Vitamin D

Vitamin D is a steroid hormone produced by skin exposure to ultraviolet light and has many important roles such as maintaining bone health via the regulation of calcium and phosphorus absorption. It also plays a role in the innate and adaptive immune system. Due to the ubiquitous reduction in the time spent outdoors, maternal HM concentrations of vitamin D (25-Hydroxyvitamin D) are often deficient. Since HM vitamin D levels are positively related to maternal serum concentrations [274,275,276] there are serious concerns regarding the vitamin D status of exclusively breastfed infants, evidenced by a resurgence in the diagnosis of rickets [277]. Maternal daily vitamin D supplementation of 400–2000 IU of vitamin D/day increases HM concentrations and subsequently infant 25-Hydroxyvitamin D status [278]. Hence, the current recommendations of the American Academy of Pediatrics is that all breastfed infants be supplemented with 400 IU/day of oral vitamin D from birth [279].

It is not clear whether vitamin D intake during pregnancy and lactation lowers the risk of infant allergies. In a number of studies, high maternal vitamin D levels have been associated with increased risk of eczema, asthma, food allergy or sensitization to food allergens [280,281,282] while others report reduced risk of allergic outcomes [269,283,284,285,286] or no relationship [286,287,288]. An interesting study on a large Finnish cohort found that maternal vitamin D intake from food was associated with reduced risk of cow’s milk allergy (CMA) while supplementation of both vitamin D and folic acid was associated with increased risk of CMA [289]; however, it is likely that other lifestyle factors have contributed to this finding. Comparisons of these studies are limited due to differences in study design, methodologies, supplementation, time of measurements, along with a lack of information regarding lactation. The other issue is the reported non-linear relationship between allergy outcomes in relation to vitamin D levels with very low and very high levels increasing the risks. The optimal level for immunological health is still to be defined and this may well differ dependent on stage in pregnancy and the age of the infant. Supplementation of lactating women and monitoring of their infants for allergy has yet to be carried out and may yield different results as seen with vitamin A. Hence, due to the limited and conflicting evidence, the World Allergy Organization has not recommended supplementing women in pregnancy or lactation as an allergy preventative strategy [290].

#### 3.4.4. Iron

Iron levels in HM are relatively low (0.3 mg/L), but this micronutrient is highly bio-available to the infant with absorption rates ranging between 16% and 50%, which is higher than that available from formula feeds [291]. The reported prevalence of iron deficient anemia is <2% up to 6 months and 2–3% between 6 and 9 months in European infants [291]. Therefore, infant supplementation is generally not recommended in the first 6 months of life with the exception of infants of diabetic mothers and low birth weight infants that have low iron stores [266,292]. However, it is recommended that the first complementary foods are rich in iron [293]. A recent study has found as many as a third of healthy fully breastfed infants are iron deficient or have iron deficiency anaemia at 5 months of age [294]. Supplementation of breastfed infants (1–6 months) with 7.5 mg per day of ferrous sulfate resulted in higher haemoglobin concentration and higher mean corpuscular volume at 6 months of age than those not supplemented [295]. Better visual acuity and greater Bayley Mental and Psychomotor Developmental Indices were also recorded at 13 months in supplemented infants. Thus, the American Academy of Paediatrics recommends that exclusively breastfed term infants and those receiving more than half of their daily feeds as breast milk be supplemented with oral iron at 1 mg/kg per day from 4 months of age [296].

Adequate iron is essential for both normal infant neurodevelopment [266] and immune protection yet is the most common global micronutrient deficiency worldwide [297] with infants and children at high risk due to the high demand for rapid growth. Very few studies have investigated the relationship of infant iron status and immunological outcomes with one case-control study showing no difference in infant status with respect to eczema [298].

#### 3.4.5. Zinc

Infants and children have high requirements for zinc due to rapid growth and tissue synthesis. Zinc deficiency is not uncommon (>20%) particularly in infants/children less than 5 years of age [299,300]. Symptoms of zinc deficiency include growth retardation, altered immune function and gastrointestinal effects such as diarrhea. Those infants/children at highest risk are those consuming a combination of breast milk and a predominantly plant-based diet of low zinc content as well as prematurity and low birth weight. [301]. HM zinc content is not related to maternal zinc status and in developed settings, zinc intake from HM is considered adequate provided the mother is able to generate enough milk for her infant [301]. However, infant zinc supplementation is often indicated in low resources settings and those where complementary foods are low in zinc [301]. 

Again, research into the relationship between infant zinc intake during lactation and allergy is scarce. Of note a case control study has shown that zinc status is lower in those infants with eczema compared to their matched controls [298], which is more likely a direct effect on skin barrier rather than immune responses.

#### 3.4.6. Summary

Micronutrients are important part of the HM composition, but there is a only small body of evidence that their intake during early life may be related to allergy. In order to establish firm relationships future research will need to consider sampling and measurement methods of HM. This includes importance of adjustment for timing—pregnancy vs. lactation; foetal and infant growth trajectories; and includes better clinical outcome definition. It is also possible to measure dose (rather than concentration) by employing methods such as test weighing [302] to further improve the quality of subsequent studies.

### 3.5. The New Frontier: Human Milk Glycoproteins and Metabolites

Metabolomics is one of the newest “omics” sciences which has been integrated into HM study using a top-down systems biology approach to explore and unravel the genetic-environment-health paradigm [303]. Metabolomics, or the study of metabolites, is useful to elucidate the complex interactions of HM constituents, and to understand the physiological state of HM in various stages of lactation [304] and in response to infection. Metabolomics, together with other the “omics” such as proteomics and glycomics and genomics can enable us to understand this complex and dynamic relationship. Several complementary analytical platforms such as nuclear magnetic resonance (NMR), capillary electrophoresis (CE), liquid or gas chromatography (LC or GC) coupled with mass spectrometry (MS) have been used to profile the composition of HM [305,306]. Recent study by Andreas et al. has identified 710 metabolites in HM using various modified extraction methods, such as Folch extraction and single-phase extraction using methanol and methyl *tert*-butyl ether (MTBE) [305].

Besides characterizing the HM metabolome, temporal changes in metabolites across stages of lactation can be tracked to demonstrate the adaptation of breasts to meet the nutritional and developmental requirements of the growing infant. Using LC- and GC-MS methods, Villasenor et al. reported increases in several fatty acids such as linoleic and oleic acid, from the first to the fourth week postpartum in full-term infants, while cholesterol, fucose and α-tocopherol levels declined [306]. In NMR-based analyses, Wu et al. reported decreases to phosphocholine and glycerol-phosphocholine concentrations after the first month of lactation that coincided with an elevation in levels of choline, a compound essential for the neonate’s growth and neuronal development [307]. Whereas, Sundekilde et al. characterized and compared 51 metabolites including HMOs, in preterm and full-term milk up to 100-days post-partum [304,308]. Lacto-*N*-difucohexaose I, 3′-sialyllactose and 6′-sialyllactose were identified to be higher in preterm milk compared to term milk [304]; these HMOs have been implicated in the onset of necrotizing enterocolitis in rat pups [309] and infants [310]. Recent studies have revealed strong associations between HM metabolites (including HMOs) and the microbiota of the infant’s gut [311]; this content was covered in earlier sections of this review.

The hygiene hypotheses have expanded our understanding of how allergic disease originates during infancy. Equally important and likely in response to our microbial environment is the role of breastfeeding in promoting tolerance to antigens and subsequently reducing the incidence of allergy and asthma [312,313]. This protection is potentially related to bioactive compounds such as secretory immunoglobulin A (sIgA) and TGF-β, present in colostrum and mature human milk that provide protection during the time when the infant’s own immune responses are immature. TGF-β is discussed in the earlier sections of this review and this section will focus on a few constituents of HM in relation to infant infection and inflammation as follows: 2 glycoproteins, secretory immunoglobulin A, and lactoferrin, and low molecular weight compounds such as lactose, choline and anti-inflammatory short-chain fatty acids. Increasingly, we are appreciating the anti-infective and anti-inflammatory roles of HM microbiota to directly influence the infant’s gut microbiome, and of HMOs which drive the growth of microbes to shape gut immunity. These interactions between HM metabolites, the gut microbiome and allergic disease are reviewed in more detail by Kumari and Kozyrskyj [314] and Julia et al. [315]. The expanded role for antimicrobial proteins/peptides in HM, as breakdown products of lactoferrin, will only be briefly mentioned in this section.

#### 3.5.1. Secretory Immunoglobulin A (sIgA)

Secretory Immunoglobulun A (sIgA) is the principal immunoglobulin on human mucosal surfaces which blocks microorganisms and toxins from attaching to mucosal epithelial cells. While oral administration of monoclonal antigen-specific IgA prevents infection with bacterial and viral pathogens, in its natural polyclonal state, non-specific sIgA protects against gastrointestinal and respiratory infections [316]. In colostrum, levels of non-specific sIgA reaching 12 g/L are not uncommon, and they decrease to 1 g/L in mature milk [317]. The HM transfer of sIgA from mother to an infant provides protection against infection by binding pathogens and stimulating gut microbes until the infant immune system takes over to produce sufficient sIgA levels [318]. It also has an important role in the development of oral tolerance to gut microbiota. Fecal sIgA concentrations reach a peak of 4.5 mg/g feces at 1 month of age in exclusively breastfed infants (fed some formula immediately after birth); they decline to 1.5 mg/g of feces at 5 months of age where they remain for the duration of infancy [319]. In exclusively formula-fed infants, however, fecal sIgA concentrations peak at 1.5 mg/g feces, drop to 1 mg/g feces at 3 months, then reach comparable levels to breastfed infants at 9 months of age. Much higher sIgA levels have been observed 1 week after birth with exclusive breastfeeding [320]. Low levels of non-specific faecal IgA in infants were among the first associated with a higher risk of allergy [321].

The production of intestinal IgA commences around 1 month after birth when low levels of fecal sIgA can be detected in non-breastfed infants [322]. Hence, sIgA in colostrum has been likened to an immune booster, a beneficial attribute that varies by maternal characteristics and can be impacted by medical intervention. Residual country variation in colostrum sIgA levels has been reported, even after accounting for collection time, and maternal parity, smoking, fruit and fish consumption, and allergen sensitization [323]. Cesarean delivery was independently associated with reduced sIgA colostrum levels in this study. Breakey et al. reported lower HM sIgA levels in time periods before and after respiratory or gastrointestinal infections in 8-month old infants of a traditional population living in rural Argentina [324].

As evident by the presence of fecal IgA in exclusively formula-fed infants, full-term infants produce substantial levels of their own IgA within 3 months after birth [325]. However, the highest IgA levels are seen in exclusively breastfed infants and they increase in direct proportion to the “dose” of HM (exclusive, partial versus no breastfeeding) provided to the infant. At this age, the likelihood of *C. difficile* colonization in gut microbiota was reduced by 75% among infants with fecal IgA levels [326] in the highest tertile, independent of parity, birth mode and breastfeeding status. While *C. difficile* presence in the infant gut is not uncommon, it is a marker for lowered colonization resistance to pathogenic bacteria and has been found to be associated with future allergic disease [327,328]. Hence, BM and infant sIgA have an important role in reducing *C. difficile* colonization. Furthermore, infant fecal IgA levels are noted to be inversely associated with infant serum levels of IgE and lower binding of IgA to *Bacteroides* species increases risk for asthma at age 7 [227,321].

#### 3.5.2. Lactoferrrin

Lactoferrin is a large molecular weight glycoprotein that is also present in colostrum and transition milk, and at higher levels than in mature milk [329]. Lactoferrin participates in host defense against microbial pathogens by binding bacterial membranes, binding iron and making it less available for microbial growth, down-regulating tumor necrosis factor-alpha (TNF-α) and interleukin-1β (IL-1β) production, and stimulating the maturation of lymphocytes [330]. Peptide breakdown products of lactoferrin have specific direct antibacterial and antifungal activity.

Higher lactoferrin levels were seen in HM preceding and following an infectious episode in the rural infants of the Breakey et al. study [324]. Since this association with infection was in the opposite direction to that seen for sIgA secretion in the same infants, the study authors proposed that lactoferrin “responds” to an infection. Lactoferrin is detected in infant feces. Mastromarino et al. found fecal *bifidobacteria* and *lactobacilli* concentrations in newborns to be positively correlated with fecal lactoferrin levels soon after delivery [329]. Due to reported associations between child atopy with reduced and not elevated *lactobacillus* abundance in the infant gut [331], it is interesting that Zhang et al. found eczema and atopic sensitization at 6 months (but not later) to be more likely in infants of mothers with higher HM levels of lactoferrin at 6 weeks after birth [332]. Upper respiratory tract infections were less likely when children were 1 or 2 years of age with higher HM lactoferrin. Clearly, the interactions between anti-infective and anti-inflammatory effects of this HM protein are complex and require further study.

#### 3.5.3. Low Molecular Weight Metabolites

##### Milk Fatty Acids

Milk lipids are principal macronutrients in HM and account for over 50 % of the infant energy daily intake requirements. Polyunsaturated fatty acids (PUFAs), more specifically the omega-3 (ω-3) fatty acids: docosahexaenoic (DHA) and eicosapentaenoic (EPA), have been shown to have anti-inflammatory effects in chronic inflammatory diseases, such as asthma [333]. Several specialized pro-resolving mediators such as resolvin and protectin, are synthesized from ω-3 fatty acids by lipoxygenase and cyclooxygenase in Th2-cytokine-stimulated macrophages and airway epithelial cells of human and murine origin [334,335]. These mediators have anti-inflammatory properties and demonstrated suppressive effects on allergic asthma [336].

More recently, the short-chain fatty acids (SCFAs), acetate, butyrate and propionate, have gained interest as mediators of allergic inflammations. They are produced by gut microbes and are used as an energy source by gut epithelial cells (colonocytes) and after absorption, by the liver for gluconeogenesis [314]. Increasingly, inflammation is being viewed as a by-product of the metabolic activity of gut microbiota from evidence that SCFAs are altered in children who are or become overweight or atopic. New evidence shows that maternal SCFA levels during pregnancy can directly impact the health of infants. Thorburn et al. observed that when a high-fibre diet was consumed during pregnancy, maternal serum acetate (but not other SCFA) levels were higher [337]. Lower serum levels of acetate during pregnancy were associated with wheeze in infants. In a follow-up murine model experiment, feeding dams acetate during pregnancy and the immediate postpartum period reduced the development of allergic airway inflammation in offspring.

SCFAs are the first metabolites produced by the gut microbiota of newborns, with synthesis increasing rapidly after birth [338]. In the few published studies, total SCFA levels are elevated in the gut of formula-fed versus breastfed infants born at term gestation, yet relative to other SCFA, acetate levels are highest with exclusive breastfeeding [314,339]. Since microbiota have been detected in HM and breastfeeding influences SCFA levels in infants, it is quite plausible that HM contains SCFA. In our pilot comparison of HM across 5 countries, butyrate and acetate were detected by NMR spectroscopy in HM collected 1 month after vaginal delivery in women who had not received antibiotics. Tan et al. have observed a reduction in food allergy and total serum IgE levels in mice treated with acetate and butyrate, but not propionate in drinking water [340]. This protection against food allergy was not observed in the absence of gut microbiota, suggesting that in addition to SCFAs, a cascade of other signaling molecules are required to prevent sensitization to food antigens [341].

##### Choline

Choline is a component of the non-protein nitrogen in human milk and is an important metabolite for lipid synthesis and in the neurodevelopment of the infant [342]. The circulatory concentration of free choline, phosphocholine, glycerophosphocholine in breastfed infants is positively correlated with the choline contents of consumed HM [343]. Ozarda et al. has demonstrated that the water-soluble choline content of early HM at 1 to 3 days postpartum was positively associated with maternal serum C-reactive protein (CRP) levels [344]. Since serum CRP is typically elevated during active infection or acute severe inflammatory processes [345,346], the Ozarda study suggests that HM choline content is a response to low-grade inflammation in the nursing mother. In fact, higher intake of dietary choline in adults has been independently associated with a reduction in inflammatory markers, namely with lowered levels of serum CRP, interleukin-6 (IL-6) and tumour necrosis factor-α (TNF-α) [347], although the exact cause of this association remains unclear. Of interest, in the Ozarda et al. study, both HM levels of choline and serum CRP were higher after caesarean versus vaginal delivery, differences which could not be attributed to the weight, height or body-mass index of breastfeeding women [344]. It is of particular interest considering known notable associations between delivery by C-section and increased risk of allergic diseases development [348].

##### Lactose

Lactose is the main component of the carbohydrate portion of HM and induces innate immunity by up-regulating gastrointestinal antimicrobial peptides that protect the infant’s gut against pathogens and regulate gut microbial homeostasis [349]. As such, the lactose concentration in HM increases after closure of the tight junctions at the initiation of lactation [350]. Before the infant can absorb lactose for energy use, it is broken down to glucose and galactose by β-galactosidase lactase in the small intestine [351]. Infant lactose intolerance is not common, as lactase is tightly regulated in infant and is then progressively down regulated in most children by 2 to 3 years of age [317]. As lactase activity decreases, the lactose moiety remains intact and then reaches the large intestine, where it is metabolized by gut microbes. This fermentation process produces hydrogen, methane, carbon dioxide and lactate [352], molecules which have the potential to cause bloating, abdominal cramps, nausea and symptoms typical of lactose intolerance. This lactose-lactase system is suggested to act as a biological timer, controlling birth spacing in human and eventual weaning. Noteworthy is that lactase deficiency is more prominent in those of Asian, South American and African descent [317]. However, there is no high-quality research providing a link between the lactose and allergic diseases development.

## 4. Breastfeeding/Human Milk Research Unmet Needs

Hitherto in this state-of-the-art review two predominant approaches were presented in the field of breastfeeding and HM research, with some studies assessing the impact of breastfeeding on health outcomes, while others testing putative associations between HM composition and the development of non-communicable disease in general, and allergy in particular. There is an evident lack of studies combining both, which therefore does not elaborate on the reasons for breastfeeding being beneficial in some children, with no effect, or even conferring a higher risk of allergy development, in the others. Furthermore, each field of study had its own methodologic challenges.

We pointed out that various methods of quantifying breastfeeding (exclusive breastfeeding versus supplementation, feeding duration categorization), differences in assessing outcomes (self-report versus clinical measures), and the timing of outcome assessment have all contributed to the inconsistent results of association studies and intervention trials. In addition, the role of breastfeeding may vary in importance depending on characteristics of the infant, such as age and mode of delivery. When comparing the impact of breastfeeding on gut microbiota in neonates versus infants, Levin et al. reported greater variance in microbial composition explained by breastfeeding at 6 months than at 1 month of age [353]. Before 3 months of age, the impact of caesarean section (CS) on gut microbial composition has been found to be stronger than breastfeeding [353,354] and to be independent of breastfeeding status [353,355]. On the other hand, findings from the Canadian Healthy Infant Longitudinal Development birth cohort suggest that early breastfeeding may modify intrapartum antibiotic prophylaxis and CS-associated dysbiosis of the gut microbiome later in infancy [355]. Hence, in addition to correctly applying breastfeeding definitions, separating ante- from postnatal influences, better phenotyping allergy outcomes and utilizing “big data” to study the impact of clusters of HM components, assessment of infant subgroups is required for a more precise recommendations to be made about breastfeeding according to maternal characteristics (i.e., asthmatic status, allergic history, parity, etc.) and delivery mode.

**Figure 1:**
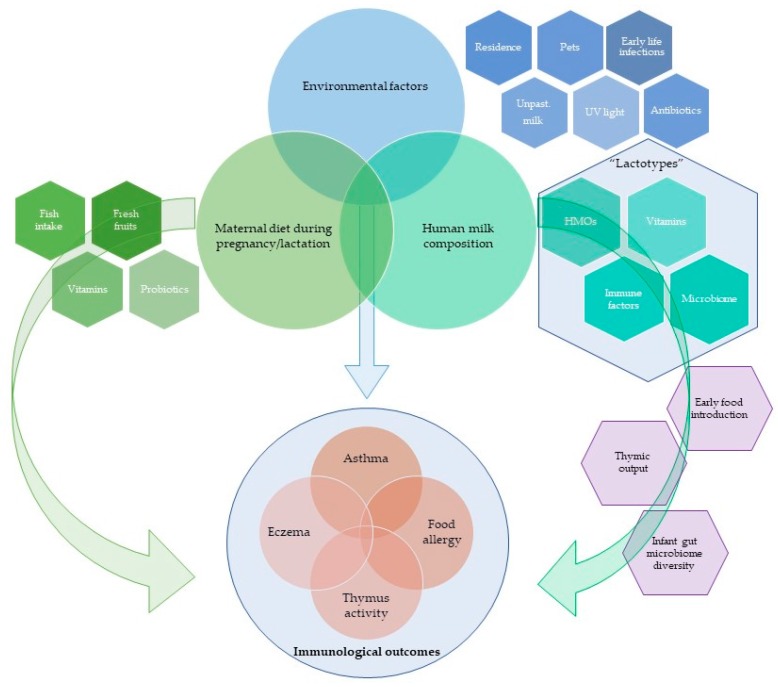
Maternal, environmental and human milk composition factors influence on immunological outcomes in child.

We have also shown that HM has a very complex composition, consisting of a wide range of immunologically active markers, oligosaccharides, live microorganisms, micronutrients, metabolites and many other bioactive compounds. Human milk composition is dynamic and variable. Early milk is particularly rich in its constituents and they undergo rapid change during the very first days of life. Country differences are also apparent, but not fully explainable at this stage, indicating that women living in different geographic locations may have distinct human milk profiles. The impact of HM composition on allergic disease development in children is still a matter of discussion as studies continue to produce conflicting results. In view of the vast number of crucial components in human milk, investigation of a single or limited range of constituents may well lead to confusing outcomes. An appealing thesis is that lactating women can be characterized according to specific and individual constituents of their milk, called “lactotypes”. Future studies should investigate the possibility of a lactotype phenotype in a large number of nursing women by analyzing human milk for multiple constituents at a time and looking for associations with a variety of immunologic phenotype and outcomes (Figure 1).

Methodological differences in the detection of constituents are also a major issue in HM studies, which makes it challenging for meta-analyses to be undertaken. HM composition comparisons between populations or countries should consider strict harmonisation of sampling, storage and analysis protocols, especially for the timing of sampling, and the collection of samples from lactating women with similar characteristics. Such studies would reduce variations caused by differences between populations and between sampling methods, although variation in storage time of milk samples could not be controlled in this way.

There are number of unmet research needs in breastfeeding and HM research (Box 1) which have arisen during the development of this manuscript and should be addressed in the future research. Addressing these needs would lead to a better understanding of the links between breastfeeding/HM composition and allergic disease development in infancy and childhood.

Box 1. **Unmet research needs in breastfeeding and human milk research.**

➢ Large and well-standardised studies of HM composition (integrated data on immune markers, HMOs, PUFAs, microbiome and metabolites), defining lactotypes and assessing variation between women residing in different countries
➢ Application of omics approaches (metabolomics, proteomics, genomics, etc.) to highlight the most important components of HM in relation to allergic diseases
➢ Studies evaluating biological activity of a specific components within HM
➢ Randomised trials of breastfeeding interventions with long-term follow-up for allergic disease development
➢ Randomised trials of early weaning (3–4 months) using different dietary approaches
➢ Large cohort studies which combine assessments of breastfeeding influence on allergy development with the constituent analysis of HM samples
➢ Development of a new intervention strategies for HM composition modification and indirect preventative effect on allergy prevention
➢ Relevance of a geographical location/lifestyle/diet and its’ influence on the composition of human milk should be assessed in more detail and research should account for these important confounders

As evidence accumulates from HM research, it will address some of these gaps to better inform policy makers, clinicians and nursing mothers. Future studies must continue to apply sound methodological approaches [356], as well as to incorporate new technologies and bring a “patient-centered” individualised approach to their application. Emerging laboratory and analytical methods will facilitate the inclusion of data on the human milk microbiome and metabolome as likely mechanistically important components of breastfeeding and these findings must be investigated for their roles in the developmental origins of health and disease (DOHAD) [154]. As allergic sensitization and allergy associated diseases are increasingly common and constitute the commonest group of common conditions afflicting young people, they provide the best opportunity to investigate DOHAD hypotheses.

## 5. Conclusions

Allergic diseases such as eczema, food allergy and asthma are the commonest chronic diseases of childhood in many countries, and there is evidence that early life events, such as variations in breastfeeding patterns, maternal diet, environmental and microbial exposures may be important in their development. There remain a number of hurdles to overcome before we come to a clear understanding on how to translate these associations into clinical practice because association is not synonymous with cause and effect. The possibility that interventions which modify maternal immunity can impact infant immune responses by changing HM composition is in part supported by associations between HM composition and immunological outcomes.

Complexity and variability in human milk composition (and known infant’s response to many of HM constituents) may also explain some of the conflicting results of studies evaluating the effect of prolonged exclusive breastfeeding and the prevention of allergic disease development. Future research needs to account for different environmental exposures and use systematic methodologies to characterize variations in human milk composition in relation to well-defined clinical and immune outcomes during childhood. Statistical approaches using cluster analysis should be implemented more frequently, in order to define the role of lactotypes, consisting of immune active molecules, PUFA’s, microbiome composition. Understanding the relationship between HM composition and development of non-communicable diseases, and particularly allergy, may allow us to establish a new paradigm in allergy prevention research—namely modulation of HM composition via maternal dietary and other interventions, in order to promote healthy infant immune development.

## Figures and Tables

**Table 1:** Maternal dietary interventions and human milk immunological composition.

Study	Intervention	Time of HM Collection Postpartum	HM Composition Changes
		***Fish Oil and Fresh Fish***	
Hawkes 2001 [119]	Fish oil supplementation	5 weeks	no significant influence on TGF-β1 and TGF-β2
Dunstan 2004 [120]	Fish oil supplementation	3 days	no significant influence on IgA and sCD14 levels
Urwin 2012 [121]	Farmed salmon supplementation	1, 5 and 28 days	no significant influence on TGF-β1, TGF-β2 and sCD14
		***Probiotics***	
Bottcher 2008 [122]	Probiotic supplementation (*L. reuteri*)	3 days and 1 month	↓ TGF-β2 and ↑ IL-10 (*borderline significance*) in 3 day samples no difference in IgA, SIgA, TGF-β1, TNF, sCD14 in 1 month samples
Prescott 2008 [123]	Probiotic supplementation (*L. rhamnosus* or *B. lactis*)	7 days	↑ TGF-β1 in HM from *B. lactis* group no significant influence on IL6, IL10, IL13, IFN-γ, TNF-α, sCD14, total IgA
Boyle 2011 [124]	Probiotic supplementation (*L. rhamnosus*)	7 and 28 days	↓ sCD14 and IgA levels in HM from *L. rhamnosus GG* group no significant influence of on TGF-β1
Hoppu 2012 [125]	Diet and Probiotic supplementation (*L. rhamnosus* and *B. lactis*)	colostrum (after birth) and 1 month	↑ IL-2, IL-4, IL10 TNF-α and total *n*-3 fatty acids in probiotic group no significant influence on IFN-γ and IL6
Kuitunen 2012 [126]	Probiotic supplementation (A combination of 2 species of *L. rhamnosus, B. breve and P. freudenreichii*)	0–3 days and 3 months	↑ IL-10 and ↓ casein IgA antibodies in probiotics group
Savilahti 2015 [127]	Probiotic supplementation (A combination of 2 species of *L. rhamnosus, B. breve and P. freudenreichii*)	0–3 days and 3 months	no significant influence on sCD14, HBD2 and HNP1–3
		***Other Interventions***	
Linnamaa 2013 [128]	Blackcurrant seed oil	after delivery and 3 months	↑ IFN-γ and ↓ IL-4 in blackcurrant seed oil group no significant influence on IL-5, IL-10, IL-12 and TNF levels
Nikniaz 2013 [129]	Synbiotic	3 and 4 months	↑ IgA and TGF-β2 in synbiotic group no significant influence on TGF-β1

“↑”—stands for increased levels of a particular factor and “↓”—stands for decreased levels of a particular factor.

**Table 2:** Human milk immunological composition and allergy development.

Study	Allergic Outcomes Assessed	Relationship between Human Milk Composition and Outcomes	
		*Human Milk Composition Factors*	*Outcome of Influence*
Kalliomaki 1999 [130]	Eczema (up to 12 months)	↑ TGF-β1 and TGF-β2 (colostrum)	higher post weaning-onset atopic disease
Jones 2002 [131]	Eczema (up to 6 months)	↓ sCD14 (3 months HM)	higher eczema incidence at 6 months of age
Bottcher 2003 [132]	Allergic sensitisation (up to 2 years) Salivary IgA (up to 2 years) Eczema (up to 2 years)	IL-4, IL-5, IL-6, IL-8, IL-10, IL-13, IL-16, IFN-γ, TGF-β1, TGF-β2, RANTES, eotaxin or SIgA (colostrum and 1 month HM)	no significant influence on atopy and/or allergy
Oddy 2003 [133]	Asthma-like symptoms (up to 12 months)	↑ TGF-β1 (2 weeks HM) TNF-α, sCD14 and IL10 (2 weeks HM)	lower risk of wheeze in infancy no significant association with infant wheeze
Savilahti 2005 [134]	Allergic sensitisation (up to 4 years) Eczema (up to 4 years)	↓ IgA casein antibodies and sCD14 (colostrum)	higher incidence of atopy development
Snijders 2006 [135]	Eczema (up to 12 months) Allergic sensitisation (up to 2 years) Wheezing (up to 2 years)	TGF-β1, IL-10, IL-12 and sCD14 (1 month HM)	no significant influence on any of the atopic manifestations
Bottcher 2008 [122]	Allergic sensitisation (up to 2 years) Eczema (up to 2 years)	↓ TGF-β2 (colostrum)	lower incidence of sensitisation during the first 2 years of life a trend of protective effect on eczema development
Kuitunen 2012 [126]	Allergic diseases (up to 5 years) Eczema (up to 5 years) Allergic sensitisation (up to 2 years)	↑ TGF-β2 (3 month HM) IL-10 and TGF-β2 (3 month HM)	higher risk of allergic disease and eczema at 2 years of age no significant association with allergic outcomes at 2 and 5 years of age
Soto-Ramirez 2012 [136]	Asthma-like symptoms (up to 12 months)	infants in the highest quartile of IL-5 and IL-13 (2 weeks HM)	higher risk of asthma-like symptoms development
Ismail 2013 [137]	Eczema (up to 12 months) Allergic sensitisation (up to 12 months)	TGF-β1, sCD14, total IgA (7 and 28 days HM)	no significant association with any of the atopic manifestations
Orivuori 2014 [138]	Eczema (up to 4 years) Asthma (up to 6 years) Allergic sensitisation (up to 6 years)	↑ sIgA (2 months HM) TGF-β1 (2 months HM) sIgA (2 months HM)	lower eczema incidence up to the age of 2 years no significant association with the outcomes no significant association with atopy or asthma up to the age of 6
Savilahti 2015 [127]	Allergic diseases (up to 5 years)	↑ sCD14 (3 months HM)	higher incidence of allergic sensitisation and eczema
Jepsen 2016 [58]	Eczema (up to 3 years) Recurrent wheeze (up to 3 years)	↑ IL-1β (1 month HM) CXCL10, TNF-α, CCL2, CCL4, CCL5, CCL17, CCL22, CCL26, TSLP, IL17, CXCL1, CXCL8, TGF-β1 (1 month HM)	lower eczema incidence up to the age of 3 years no significant association with eczema or wheeze
Munblit 2017 [139]	Eczema-like symptoms (up to 6 months) Wheeze (up to 6 months) Food allergy parental-reported (up to 6 months)	↑ TGF-β2 (1 month HM) detectable IL-13 (colostrum) detectable IL-13 (1 month HM) HGF, TGF-β1, TGF-β3, IL-2, IL-4, IL-5, IL-10, IFN-γ, IL-12 (colostrum and 1 month HM)	higher risk of eczema lower risk of food allergy lower risk of eczema no significant association with eczema, wheeze or food allergy

“↑”—stands for increased levels of a particular factor and “↓”—stands for decreased levels of a particular factor

**Table 3:** Human milk micronutrients known to be influenced by maternal diet. The range of mean concentrations is given for mature milk. Reference [241]—”Handbook of Milk Composition” summarises milk composition up to approximately 1993.

Component Affected by Maternal Diet	Concentration	Component Unaffected by Maternal Diet	Concentration
**Fat Soluble Vitamins**			
K	0.12–0.98 ug/dL [241,242,243]	Tocopherol (vit E)	207–366 ug/dL [244,245,246]
D	0.008–0.62 ug/dL [242,246,247,248]		
Retinol (Vit. A) *	40–485 μg/L [242,245,249]		
**Water Soluble Vitamins**			
Thiamin (vit B-1)	21.1–228 ug/L [249,250,251]	Folate	53–133 ug/L [241,252,253]
Riboflavin (vit B-2)	0.03–0.35 mg/L [249,251]		
Niacin (vit B-3)	68.7–260 ug/L [251,254]		
Vit B-6	0.06–0.31 mg/L [241,249,251,255]		
Cobalamin (vit B-12)	85–970 ng/L [249,255,256]		
Ascorbic acid (vit C)	35–105 mg/L [241,246,249]		
Pantothenic acid (vit B-5)	2.0–2.5 mg/L [241,251]		
Choline	144–258 mg/L [241,257]		
**Minerals**			
Selenium	3–60 ng/mL [241,249,258,259]	Zinc	0.68–12 ug/mL [241,245,260,261,262]
Iodine	9–250 ug/L [241,249,263,264,265]	Copper	0.006–0.5 ug/mL [241,245,253]
		Iron	0.3–0.9 ug/mL [245,262,266]
		Calcium	259–300 mg/L [241,245,262]
		Phosphorus	130–170 mg/L [241,245,262]
		Magnesium	30.5–31.4 mg/L [241,245]
		Sodium	111–300 mg/L [241,245,262]
		Potassium	380–630 mg/L [241,245,262]
		Chromium	0.15–0.8 ng/mL [241,247,253]
		Chloride	453–690 mg/L [241,262]
		Manganese	0.33–125 ng/mL [241,245,253,262]

* Vit.—Vitamin.
